# Are We Ready to Offer Endovascular Thrombectomy to All Patients With Large Ischemic Core?

**DOI:** 10.3389/fneur.2022.893975

**Published:** 2022-04-13

**Authors:** Presaad Pillai Perianen, Bernard Yan

**Affiliations:** Melbourne Brain Centre, Royal Melbourne Hospital, Parkville, Melbourne, VIC, Australia

**Keywords:** ischemic core, Alberta Stroke Programme Early CT Score (ASPECTS), DWI-ASPECTS, endovascular thrombectomy (EVT), CT perfusion (CTP)

## Introduction

We have read with great interest the recently published randomized controlled clinical trial, Endovascular Therapy with Acute Stroke with a Large Ischemic Lesion (RESCUE-Japan LIMIT) (1). The investigators included patients with large vessel occlusions (either intracranial internal carotid artery or middle cerebral artery M1 segment) and large ischemic core on imaging based on the Alberta Stroke Program Early CT Scores (ASPECTS) of 3–5 on either CT or MRI diffusion-weighted imaging (DWI). Patients with onset of symptoms up to 24 h were included in the study if there was diffusion-weighted imaging-fluid attenuated inversion recovery (DWI-FLAIR) mismatch, defined as diffusion restriction on DWI without signal change on FLAIR, suggesting an acute ischemic stroke (1). DWI-FLAIR mismatch was used as a surrogate marker for early onset ischaemia in the DWI-FLAIR mismatch for identification of patients with acute ischemic stroke within 4.5 h of symptom onset (PRE-FLAIR) study (2) and in Efficacy and Safety of MRI-Based Thrombolysis in Wake-Up Stroke (WAKE-UP) trial (3). In the RESCUE-Japan LIMIT trial, ~87% of patients in the endovascular thrombectomy (EVT) arm and 85% in the medical care arm used DWI-ASPECTS, and the remainder used CT-ASPECTS. There was a significantly higher proportion in the EVT arm (31%) than in the medical care arm (12.7%), achieving a modified Rankin scale (mRS) score of 0–3 [relative risk, 2.43; 95% confidence interval (CI), 1.35–4.37; *p* = 0.002] (1). However, two critical questions remained unanswered: (1) Are the CT-ASPECTS and DWI-ASPECTS scoring systems interchangeable? (2) Is the DWI-ASPECTS scoring system an appropriate substitute for volumetric analysis of ischemic core in patients with large vessel occlusion?

## Are CT-Aspects and DWI-Aspects Interchangeable?

ASPECTS has been widely used to assess the extent of early ischemic changes on CT brain for acute stroke treatment since it was published in the year 2000 (4). It is a visual assessment of areas of acute ischemic changes characterized by parenchymal hypoattenuation, loss of gray–white differentiation, and focal brain swelling over the middle cerebral artery territory, and is scored based on a 10-point scoring system, each corresponding to a particular anatomical region (4, 5). These early ischemic changes represent cytotoxic oedema and the assumed establishment of irreversible injury (4). However, the inter-rater agreement for CT-ASPECTS is uncertain. Van Seeters et al. (6) reported that the inter-rater and intra-observer agreement for CT-ASPECTS was poor and moderate [Kappa (κ): 0.219 and 0.595, respectively], and Mitomi et al. (5)reported a poor inter-rater agreement for CT-ASPECTS [Kappa (κ): 0.31].

ASPECTS has been applied to MRI, known as DWI-ASPECTS, which adopts the same 10-point scoring system (7). As MRI is a more sensitive imaging modality than CT scan, diffusion-weighted imaging (DWI) DWI-ASPECTS showed higher sensitivity than CT-ASPECTS (5, 7, 8), with DWI detecting ischemic lesions significantly more frequently than CT in 8 out of 10 regions (5). Of the 10 regions, CT had the lowest sensitivity over the internal capsule (sensitivity, 0; 95% CI, 0–0.34) and the caudate head (sensitivity, 0.06; 95% CI, 0–0.3). Despite its improved sensitivity, the inter-rater agreement for DWI-ASPECTS was poor, with κ = 0.17 (0.14–0.21), but the agreement improved after dichotomization of DWI-ASPECTS (0–5 vs. 6–10 or 0–6 vs. 7–10) to a fair level [κ = 0.62 (0.48–0.75) and 0.68 (0.55–0.79), respectively] (9). Overall, there was a poor correlation between DWI and CT-ASPECTS (*r* = 0.51) (5).

The authors of RESCUE-LIMIT referenced the SAMURAI study (10), which concluded that in hyperacute stroke, a discrepancy of 1 point between DWI-ASPECTS and CT-ASPECTS existed. Therefore, DWI-ASPECTS patients with a score of 3–4 in the study may have CT-ASPECTS of 5. REVASCAT was an example of a trial that corrected for this 1-point difference in their cohort of patients for the exclusion criteria of a large ischemic core with ASPECTS <7 and DWI-ASPECTS <6 (11). However, in the SAMURAI study (10), the interquartile range for DWI-ASPECTS was 6–9 and for CT ASPECTS was 8–10, effectively excluding ASPECTS of 3–5 (the target cohort in RESCUE-Japan LIMIT). In summary, there are pitfalls in assuming the equivalency of CT-ASPECTS and DWI-ASPECT.

## Is DWI-Aspects an Appropriate Substitute for Volumetric Analysis of Ischemic Core?

The Highly Effective Reperfusion evaluated in Multiple Endovascular Stroke (HERMES) collaborators' meta-analysis analyzed 5 foundational thrombectomy trials and demonstrated significant reduction in disability at 90 days in the thrombectomy arm compared with the medical treatment arm (adjusted OR, 2.49; 95% CI, 1.76–3.53; *p* < 0.0001) (12). On the question of ASPECTS, there was a favorable outcome for both ASPECTS 6–8 [OR, 2.36 (95% CI, 1.68–3.26)] and ASPECTS 9–10 [OR 2.66 (95% CI, 1.61–4.4)] but no significant benefit for the 121 patients with ASPECTS 0–5 [OR of 1.24 (0.62–2.42)] (12). The Systematic Evaluation of Patients Treated with Neurothrombectomy Devices for Acute Ischemic Stroke (STRATIS) registry looked at the impact of age in 57 patients with CT-ASPECTS 0–5 who underwent thrombectomy. Successful reperfusion (≥TICI 2b) was achieved in 85.5% (47/55) in the ASPECTS of 0 to 5 group; however, functional independence was only achieved in 28.8% (15/52), with no patients >75 years of age achieving good outcomes (13). This implies that a more precise method of patient and imaging selection is required in patients with expected large core infarcts.

An observational study on 59 patients looked at the accuracy of ASPECTS and CT perfusion core volume to detect an acute DWI core infarct of ≥70 ml. A CT-ASPECTS score of <7 was determined to be a cutoff for DWI core volume of ≥70 ml (sensitivity 0.74, specificity 0.86, Youden J = 0.6), and CT perfusion core volume cutoff was ≥50 ml (sensitivity 0.86, specificity 0.97, Youden J = 0.84) (14). A CT perfusion volume of ≥ 50 ml was highly specific in predicting DWI lesion volume >70 ml, which resulted in 93% of patients being correctly classified. CT-ASPECTS doubled the number of patients who were erroneously classified as small volume infarcts compared to CT perfusion (14). Kim et al. retrospectively analyzed patients who underwent EVT based on DWI-ASPECTS and divided them into 2 groups (DWI ASPECTS 4–6 vs. 7–10). They demonstrated a good outcome in both groups, An outcome of 61/120 in those with DWI-ASPECTS > 6 and 20/51 in those with DWI-ASPECTS 4–6, and the difference between the 2 groups was not significant (15). The implication is that high DWI-ASPECT probably correlates to a small ischemic core resulting in good outcomes with intervention. However, outcomes of DWI-ASPECTS < 7 possibly vary widely.

Endovascular thrombectomy studies restricting the inclusion of patients with a core volume <70 ml showed a good outcome in the thrombectomy arm (16, 17). The Diffusion-Weighted Imaging or Computerized Tomography Perfusion Assessment with Clinical Mismatch in the Triage of Wake Up and Late Presenting Strokes Undergoing Neurointervention (DAWN) trial showed a significant difference between EVT (5.5 ± 3.8) and control group (3.4 ± 3.1) in the utility-weighted modified Rankin score at 90 days [adjusted difference of 2.0 (95% CI, 1.1–3)]. In the subgroup analysis of the 2 groups with patients aged <80 years, one with a core infarct volume <31 ml and the other with a core infarct volume 31–51 ml, the adjusted difference for the former is 1.8 (95% CI, 0.6–2.9) compared to the latter with an adjusted difference of 2.5 (95% CI, −0.6 to 5.5) (16). Similar findings were seen in DEFUSE-3 for functional independence at 90 days, significantly favoring the endovascular arm compared to medical therapy with a risk ratio of 2.67 (95% CI, 1.6–4.48). The risk ratio for the subgroup of ischemic core volume of 10–25 ml was 4.4 (95% CI, 1.41–20.33) and ischemic core volume 25–70 ml was 3.06 (95% CI, 1.01–13.52) (13). These two landmark trials indicate that a selected group of patients with a core volume of <70 ml is favorable with EVT. On the other hand, there is emerging evidence that EVT in patients with a core infarct volume >70 ml has variable outcomes (18–20). In the SELECT study, three different large core volumes were assessed, which were 50–70, 70–100, and >100 ml comparing EVT and medical management. Endovascular thrombectomy in the 50–100 ml groups demonstrated favorable outcomes in 8 of 29 patients, whereas no patient (0 of 10) with a core volume >100 ml had functional independence at 90 days (18). The odds of functional independence showed a 42% reduction per 10 ml increase in core volume with an adjusted OR 0.58 (95% CI, 0.39.87) and *p* = 0.007 (18). Chen et al. also looked at a favorable outcome (mRS 0–2 at 90 days) in patients with a core volume ≥70 ml who underwent EVT compared to those with intravenous thrombolysis. Patients in the EVT arm were more likely to achieve mRS 0–2 compared to IVT (OR, 3.875; 95% Cl 1.068–14.055, *p* = 0.039) after adjusting for age, National Institutes of Health Stroke Scale (NIHSS), time from onset to imaging, and ischemic core volume. Their data concluded that 31% of patients with an ischemic core volume of >70 ml who underwent EVT attained a favorable outcome (19). Yoshimoto et al. concluded that the treatable upper ischemic core limit was up to 120 ml in selected patients, and that patients who underwent EVT with a core volume of 70–100 ml still showed benefit (20). This demonstrates that there is a constant push to establish the ceiling of core volume with an overall aim of improving patients' quality of life with minimal complications. As there is strong evidence that ischemic core volume is directly correlated to outcome, the critical question is whether DWI-ASPECTS can serve as a surrogate marker for ischemic core volume.

De Margerie-Mellon et al. demonstrated that DWI-ASPECTS showed a reasonable correlation with ischemic core volume (ρ = −0.82; 95% CI, −0.86 to −0.77; *P* < 0.001); however, it was only in patients with DWI-ASPECTS ≥7 and <4, which corresponded to a volume of <70 or >100 ml, respectively. Ischemic core volume was distinctly variable in DWI-ASPECTS 4–6 patients, with a median volume of 66 ml, and an interquartile range (IQR) between 41 and 97 ml (21). A more recent Japanese study also showed a highly variable range of ischemic core volume between 70 and 226 ml for DWI-ASPECTS 3–5. The large ischemic cores in this study were divided into groups A (70–100 ml) with a median of 80 ml and IQR of 74–87 ml, B (100–130 ml) with a median of 107 and IQR of 101–117 ml, and C (>130 ml) with a median of 178 ml and IQR of 175–194 ml. The median DWI-ASPECTS for group A was 5 and for group B was 6, with both of the groups sharing the same IQR of 4–6 despite having such large differences in core infarct volume. Group C had a median DWI-ASPECTS of 3; however, the IQR was 3–6, which was, again, overlapping with that of groups A and B (20). Similar findings have demonstrated that DWI ASPECTS was a reasonable substitute for ischemic core volume only when the score was ≥7. When DWI ASPECTS was <7, there was a notable variance between DWI-ASPECTS and ischemic core volume (22, 23). Therefore, DWI-ASPECTS might be an appropriate estimate for ischemic core volume when the score is ≥7. However, it is a poor substitute for ischemic core volume if DWI ASPECTS is between 3 and 6.

We also note that there are six ongoing studies on the large ischemic core (TELSA, TENSION, IN EXTREMIS LASTE, SELECT2, ANGELASPECT, and SICARIO). These studies utilize a variety of imaging methods including CT-ASPECTS, DWI-ASPECTS, and volumetric analysis as qualifying imaging markers.

## Conclusion

We would like to congratulate the RESCUE-Japan LIMIT authors on their successful thrombectomy trial demonstrating treatment benefits in patients with a large ischemic core defined predominantly by DWI-ASPECTS. However, major questions remain. First, the equivalence of non-contrast CT and MRI is unclear. In addition, MRI is not widely adopted in acute stroke care pathways, and non-contrast CT may be the only imaging modality to assess ASPECTS score. Second, DWI-ASPECTS is an unreliable surrogate marker for large ischemic core volumes. On this basis, we envisage that there will be reluctance from certain geographic regions to adopt RESCUE-Japan LIMIT criteria for thrombectomy.

## Author Contributions

PP and BY conceived and drafted the manuscript and revised the draft. Both authors contributed to the article and approved the submitted version.

## Conflict of Interest

The authors declare that the research was conducted in the absence of any commercial or financial relationships that could be construed as a potential conflict of interest.

## Publisher's Note

All claims expressed in this article are solely those of the authors and do not necessarily represent those of their affiliated organizations, or those of the publisher, the editors and the reviewers. Any product that may be evaluated in this article, or claim that may be made by its manufacturer, is not guaranteed or endorsed by the publisher.
